# Rising Geographic Variation in Alzheimer's Disease Mortality

**DOI:** 10.1093/geroni/igab046.2769

**Published:** 2021-12-17

**Authors:** Jessica Ho, Yujin Franco

**Affiliations:** 1 University of Southern California, Los Angeles, California, United States; 2 University of Southern California, University of Southern California, California, United States

## Abstract

The burden of Alzheimer’s disease (AD) mortality has increased rapidly, growing by nearly 4- (men) and 6-fold (women) between 1990-2017. Limited attention has been paid to geographic inequalities in AD mortality. This study examines age-standardized AD mortality across 10 regions and the urban/rural continuum among adults aged 65+ using National Center for Health Statistics mortality and population data. We also examine mortality for a broader category, Alzheimer’s disease and related dementias (ADRD), to address potential underreporting. The East South Central has the highest AD death rates and experienced larger increases–5-fold (men) and 7-fold (women)–than the nation as a whole. The Middle Atlantic consistently experienced the lowest AD mortality over the past quarter-century. Differences between the best- and worst-performing regions widened over time. AD mortality was 2.5 times higher in the East North Central than the Middle Atlantic region in 2017 (268 vs. 110 [men] and 374 vs. 147 [women] deaths per 100,000). Rural areas facing health care shortages and socioeconomic deprivation may encounter substantial challenges in addressing rising AD mortality. In several regions, rural disadvantages in AD mortality emerged and widened over time. The largest gaps between nonmetros and large central metros are in the East North Central, South Atlantic, and New England, as well as Appalachia (men) and West South Central (women), with nonmetros having 14-56% higher mortality than big cities. These findings identify the heavy burden of AD mortality in the Southern and rural U.S. and have important implications for health care, service, and caregiving provision.

